# Ensemble Docking, MD, and MM/PBSA Identify Flavonoids as Putative Modulators of EFNB2/B3-Nipah Virus G Interaction

**DOI:** 10.3390/ijms27146137

**Published:** 2026-07-09

**Authors:** Carlos Vargas-Echeverría, Oscar Saurith-Coronell, Olimpo Sierra-Hernandez, Juan F. Santos-Rodríguez, Juan D. Rodríguez-Macías, José R. Mora, José L. Paz, Breallan De Jesús Rómero Pájaro, German Darío Idarraga Negrete, Ricardo Olimpio de Moura, Igor José dos Santos Nascimento, Edgar A. Márquez Brazón

**Affiliations:** 1 Departamento de Medicina, División Ciencias de la Salud, Universidad del Norte, Km 5, Vía Puerto Colombia, Puerto Colombia 081007, Colombia; vargasce@uninorte.edu.co (C.V.-E.); osaurith@uninorte.edu.co (O.S.-C.); olimpos@uninorte.edu.co (O.S.-H.); 2 Grupo de Investigaciones en Química y Biología, Departamento de Química y Biología, Facultad de Ciencias Básicas, Universidad del Norte, Carrera 51B, Km 5, Vía Puerto Colombia, Barranquilla 081007, Colombia; jsantosf@uninorte.edu.co; 3 Facultad de Ingeniería, Departamento de Ingeniería de Sistemas, Universidad del Norte, Carrera 51B, Km 5, Vía Puerto Colombia, Barranquilla 081007, Colombia; 4 Facultad de Ciencias de la Salud, Exactas y Naturales, Universidad Libre, Barranquilla 080001, Colombia; 5 Grupo de Química Computacional y Teórica (QCT-USFQ), Departamento de Ingeniería Química, Universidad San Francisco de Quito, Diego de Robles y Vía Interoceánica, Quito 170901, Ecuador; jrmora@usfq.edu.ec; 6 Departamento Académico de Química Inorgánica, Facultad de Química e Ingeniería Química, Universidad Nacional Mayor de San Marcos, Lima 15081, Peru; jpazr@unmsm.edu.pe; 7 Especialización en Medicina Interna, Universidad Libre, Barranquilla 080001, Colombia; breallan-romerop@unilibre.edu.co (B.D.J.R.P.); germand-idarragan@unilibre.edu.co (G.D.I.N.); 8 Programa de Pós-Graduação em Ciências Farmacêuticas (PPGCF), Universidade Estadual da Paraíba (UEPB), Campina Grande 58429-500, PB, Brazil; ricardo.olimpiodemoura@servidor.uepb.edu.br (R.O.d.M.); igor.n@visitante.uepb.edu.br (I.J.d.S.N.)

**Keywords:** Nipah virus, viral entry modulation, flavonoids, molecular dynamics simulations, in silico scaffold discovery

## Abstract

Nipah virus (NiV) is a highly lethal zoonotic pathogen with significant pandemic potential, for which no approved antiviral therapies are currently available. Viral entry is mediated by the interaction between the NiV attachment glycoprotein (NiV-G) and host ephrin receptors, particularly ephrin-B2 (EFNB2) and ephrin-B3 (EFNB3), making this interface an attractive therapeutic target. In this study, we evaluated a set of structurally related flavonoids, apigenin, cynaroside, and lonicerin, as potential modulators of the EFNB2–NiV-G and EFNB3–NiV-G interactions. These compounds were selected based on their structural similarity, reported antiviral activity, chemical diversity, and favorable drug-like properties. Apigenin was employed as a reference scaffold due to its well-characterized pharmacological profile and its suitability for guiding analog-based compound selection. Apigenin served as a reference scaffold for selecting structurally related flavonoids, which were analyzed through density functional theory optimization, molecular docking, pharmacokinetic and toxicity predictions, molecular dynamics simulations, and binding free energy calculations. These flavonoids demonstrated high predicted affinity for both the EFNB2–NiV-G and EFNB3–NiV-G interfaces. According to results, these compounds consistently interacted with residues known to play a critical role in receptor recognition, with special emphasis on leucine and tryptophan residues within the G–H loop. These residues are well established as key determinants in the entry process of Nipah virus (NiV) into host cells, highlighting the potential relevance of these flavonoid–protein interactions. Molecular dynamics analyses indicated that flavonoid binding reduced the affinity and the conformational flexibility at the receptor–glycoprotein interfaces and decreased the stability of the complexes. Pharmacokinetic and toxicity predictions suggested favorable drug-like properties for the flavonoids, with apigenin displaying the most balanced profile. Collectively, these results support the potential of selected flavonoids as modulators of EFNB2–NiV-G and EFNB3–NiV-G interactions and provide a rationale for their prioritization in experimental studies aimed at developing scaffolds for the modulation of viral entry against Nipah virus.

## 1. Introduction

The *Henipavirus nipahense* commonly known as Nipah virus (NiV) is a zoonotic pathogen with a single-stranded, negative-sense RNA genome that was first identified during an outbreak in Malaysia in 1998. It is an obligate intracellular pathogen belonging to the family *Paramyxoviridae* and the genus *Henipavirus* [1,2,3]. Fruit bats of the genus *Pteropus* constitute its primary natural reservoir and are widely distributed across tropical and subtropical regions of Asia, Oceania, and parts of East Africa. Over the past years, recurrent outbreaks have been reported in several countries of Southeast Asia, leading to hundreds of confirmed and suspected human cases. These outbreaks have drawn attention to the virus’s exceptionally high case-fatality rate, which has been reported to reach up to 75% in certain settings [4]. Transmission occurs mainly through two routes: animal-to-human and human-to-human [5,6,7]. Human-to-human transmission has been well documented, particularly in the context of prolonged close contact or exposure to bodily fluids from infected individuals. This mode of transmission substantially increases the risk of secondary infections within households and healthcare settings. Patients infected with NiV usually present with influenza-like symptoms, including fever, headache, myalgia, vomiting, and sore throat. In a considerable proportion of cases, these initial manifestations are followed by neurological involvement, most commonly reflected by altered consciousness and clinical features consistent with acute encephalitis. In addition, some patients develop atypical pneumonia with severe respiratory complications, such as dyspnea, which may progress to acute respiratory distress syndrome [8,9]. Certain population groups are disproportionately affected and are more likely to experience severe disease. These include individuals living in endemic areas, older adults, young children, and patients with underlying conditions such as diabetes mellitus, cardiopulmonary disease, immunosuppression, malnutrition, or pregnancy. The increased susceptibility of these groups poses a sustained challenge for public health systems, particularly during outbreak situations. Many of the affected areas face significant limitations in healthcare infrastructure, which increases the risk of rapid transmission and complicates timely containment efforts. Taken together, these factors highlight the need for strengthened surveillance, improved access to rapid diagnostic tools, and effective infection prevention and control strategies to reduce the likelihood of future outbreaks and mitigate their impact [10,11]. Patients infected with NiV usually present with influenza-like symptoms, including fever, headache, myalgia, vomiting, and sore throat. In a considerable proportion of cases, these initial manifestations are followed by neurological involvement, most commonly reflected by altered consciousness and clinical features consistent with acute encephalitis [8,9]. Certain population groups are disproportionately affected and are more likely to experience severe disease. These include individuals living in endemic areas, older adults, young children, and patients with underlying conditions such as diabetes mellitus, cardiopulmonary disease, immunosuppression, malnutrition, or pregnancy. Many of the affected areas face significant limitations in healthcare infrastructure, which increases the risk of rapid transmission and complicates timely containment efforts. Taken together, these factors highlight the need for strengthened surveillance, improved access to rapid diagnostic tools, and effective infection prevention and control strategies to reduce the likelihood of future outbreaks and mitigate their impact [10,11].

**Figure 1:**
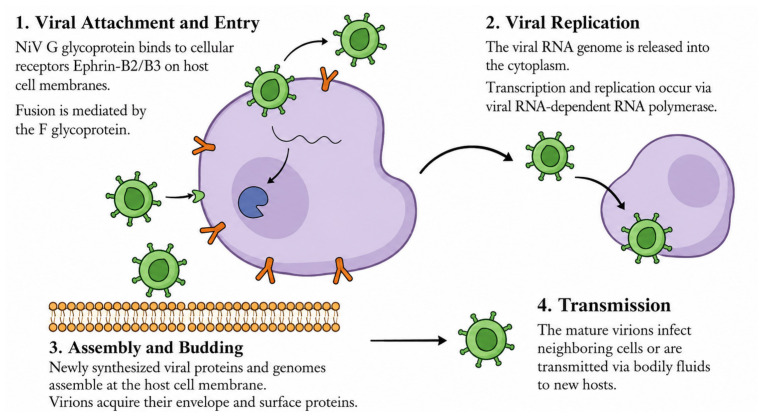
Schematic representation of the NiV lifecycle and potential points of flavonoid interference.

The Ephrin-B2 (EFNB2) and Ephrin-B3 (EFNB3) act as entry receptors for NiV through a shared molecular framework. Both proteins exhibit a high degree of sequence and structural conservation within the extracellular ephrin domain that constitutes the NiV-G binding interface, including the β-sheet core and several surface-exposed loops critical for receptor recognition. Conserved residues involved in hydrophobic contacts and hydrogen-bond interactions generate a compatible receptor–virus binding geometry in both receptors, allowing efficient viral attachment and fusion in cellular system. Viral entry is initiated by the attachment glycoprotein NiV-G, which specifically recognizes and binds these ephrin receptors, thereby anchoring the virus to the host cell membrane. This event triggers the action of the fusion glycoprotein NiV-F, which mediates the merger of the viral envelope with the host cell membrane and permits delivery of the viral RNA genome into the cytoplasm. Once internalized, NiV relies on host cellular machinery while using its own RNA-dependent RNA polymerase to drive transcription and genome replication, producing both new genomic RNA and viral mRNAs required for protein synthesis [12,13]. Newly synthesized viral RNA and structural proteins are transported to the inner surface of the plasma membrane, where virion assembly takes place. During budding, nascent particles acquire their lipid envelope and surface glycoproteins from the host cell membrane, resulting in the formation of infectious progeny virions. These mature particles are subsequently released, enabling local spread and transmission to new hosts through contact with infected bodily fluids (Figure 1). The efficiency of this replication cycle, together with the absence of approved antiviral therapies and the high case-fatality rates associated with infection, highlights the potential of NiV to pose a serious global health threat [14]. A detailed understanding of the molecular mechanisms governing viral entry, replication, and egress therefore remains essential for the identification of viable therapeutic targets and the development of effective antiviral strategies.

At present, there are no agents approved for clinical use in the treatment of NiV infection. Current management relies largely on supportive care aimed at controlling symptoms and complications, without directly targeting viral replication or disease mechanisms. This therapeutic gap represents a major unmet need, particularly given the high morbidity and mortality associated with NiV infection [15]. Several antiviral candidates have been explored in preclinical models. Favipiravir, a purine analog that inhibits viral RNA-dependent RNA polymerase (RdRp), has demonstrated selective antiviral activity in vitro and efficacy in hamster models of NiV infection. In parallel, monoclonal antibody-based approaches have emerged as promising strategies, particularly those targeting viral surface glycoproteins essential for host cell entry [15,16,17,18]. Recombinant vaccine platforms incorporating the NiV G and F glycoproteins have also shown protective effects by eliciting neutralizing immune responses and preventing viral attachment and membrane fusion. By blocking these early entry steps, such strategies interrupt the viral life cycle at its initial stage, highlighting viral glycoproteins as rational targets for entry-focused therapeutic intervention [16]. Within this context, flavonoids have attracted increasing interest due to their structural diversity, broad spectrum of biological activities, and generally favorable safety profiles. By leveraging these intrinsic properties, a novel therapeutic strategy for this viral infection can be proposed. Although several approaches have been suggested to modulate the interface between ephrin receptors and the NiV-G protein, none have specifically exploited the structural advantages of flavonoids to disrupt this interaction. Consequently, the use of flavonoid scaffolds represents a promising and underexplored avenue for targeting the virus–receptor interface. Their potential relevance is especially notable for emerging viral pathogens such as NiV, where effective, low-toxicity therapeutic options remain urgently needed, and conventional antiviral pipelines are limited.

Flavonoids are polyphenolic natural products characterized by broad target engagement profiles, reflecting their intrinsic polypharmacological nature. This capacity to interact with diverse proteins does not necessarily confer therapeutic selectivity but renders them useful molecular scaffolds for probing complex biological interfaces, including host–virus interaction systems such as those mediated by ephrin receptors EFNB2 and EFNB3. This multitarget potential is particularly relevant for NiV, whose entry relies on conserved protein–protein interactions at the host–virus interface rather than on a single viral enzymatic function. Apigenin is a well-characterized dietary flavone that has received considerable attention due to its broad-spectrum antiviral activity against both RNA and DNA viruses, including members of the *Flaviviridae*, *Orthomyxoviridae*, and *Paramyxoviridae* families. Structurally, apigenin possesses a rigid flavone core decorated with multiple hydroxyl groups, enabling a favorable balance between hydrogen bonding and hydrophobic interactions and allowing adaptability across diverse protein binding surfaces; this characteristic renders apigenin an attractive scaffold. Its capacity to interact with binding sites across a wide range of physicochemical environments highlights its potential as a suitable candidate for the design of interface-modulating molecules. From a pharmacological perspective, apigenin displays drug-like features such as moderate lipophilicity, chemical stability, and a well-established safety profile. Apigenin has been shown to exert anti-inflammatory and immunomodulatory activities through modulation of key signaling pathways, including NF-κB, MAPK, and Nrf2, which are closely implicated in virus-induced inflammation and dysregulated host responses [19,20,21,22].

In the context of emerging infectious diseases such as NiV, where experimental research is severely constrained by biosafety level 4 (BSL-4) requirements, in silico approaches represent a pragmatic and well-established strategy for early-stage antiviral discovery [23]. These computational methods allow the rapid screening of large libraries of natural and synthetic compounds, estimation of binding affinities toward viral or host targets, and preliminary evaluation of pharmacokinetic and toxicity-related parameters before progressing to resource-intensive experimental studies. Molecular docking and molecular dynamics (MD) simulations, complemented by free-energy calculations, provide mechanistic insights into the stability and specificity of ligand–receptor interactions under physiologically relevant conditions [24,25,26,27,28,29].

Notably, in silico screening has been successfully applied to identify natural inhibitors against other viral pathogens, such as SARS-CoV-2, leading to the rapid characterization of candidates such as quercetin and kaempferol with potential antiviral properties [30,31,32]. Similar computational strategies have facilitated the identification of entry inhibitors for the Ebola virus through virtual screening of FDA-approved drug libraries, as well as the discovery of lead compounds targeting the NS5 RNA polymerase of Zika virus [33,34,35]. By narrowing the candidate pool to molecules with the most favorable interaction profiles, in silico approaches significantly improve the efficiency and precision of antiviral discovery pipelines [36,37,38]. This is particularly critical when dealing with high-risk pathogens, where minimizing direct handling of live viruses and concentrating resources on the most promising candidates is both scientifically sound and ethically advantageous [39,40].

## 2. Results and Discussion

### 2.1. Data Collection

To initiate the compound selection strategy, Apigenin was chosen as the reference molecule due to its well-documented biological versatility and broad antiviral potential. Beyond its recognized antioxidant and anti-inflammatory properties, Apigenin is also known for its ability to modulate cellular signaling pathways frequently exploited by viruses, including MAPK, PI3K/AKT, and NF-κB-key regulators of early entry events and downstream replication. Its consistent activity across these pathways supports its use as a chemically reliable starting point for identifying structurally related antiviral candidates [35,36,37,38,39,40,41]. Apigenin contains a planar flavone core with hydroxyl groups capable of forming stable hydrogen bonds and pi stacking interactions, properties that enhance ligand–receptor complementarity in docking and molecular dynamics analyses. Improvements in its formulation, particularly through nanoencapsulation and phospholipid carriers, have also increased its pharmacokinetic suitability. This mechanistic flexibility, combined with a favorable structural and pharmacokinetic profile, positioned Apigenin as an ideal starting point for analog-based compound discovery. Using apigenin as a reference compound, a focused panel of eight structurally related flavonoids was curated: cynaroside, lonicerin, fisetin, diosmetin, myricetin, quercetin, taxifolin, and kaempferol. These compounds were selected not only for their structural resemblance to Apigenin but also for their reported antiviral activities, chemical diversity, and favorable drug-like properties. Their structures were obtained in both SMILES and SDF formats and served as the basis for subsequent computational analyses (Table 1).

In this framework, the selection of target proteins was carefully considered in light of the high mutability commonly observed in viral proteins. For instance, proteins such as NiV-G are prone to significant sequence and structural variations, which may alter ligand binding over time. This inherent variability can compromise the long-term effectiveness of inhibitors designed against a single viral conformation, thereby limiting their broader applicability. To address this challenge, we explored an alternative strategy focused on host receptors, specifically EFNB2 and EFNB3. These receptors play a critical role in viral entry and, importantly, exhibit greater structural stability compared to viral proteins. Their relatively conserved nature makes them attractive targets for the design of more robust therapeutic agents. The initial approach was based on the assumption that flavonoids initially bind to the receptors, suggesting that the interaction of the NiV-G viral protein may be modulated by the presence of the ligand. In particular, the presence of flavonoids at this binding interface may interfere with virus–host recognition and entry through partial disruption of the interaction between these two structures. It is important to emphasize, however, that the interaction of flavonoids with the receptor is not intended to downregulate or disrupt downstream signaling pathways mediated by ephrin receptors, but rather to transiently modulate the virus–receptor interface. We recognize, however, that targeting host proteins raises important considerations regarding druggability and the potential for off-target effects or toxicity. This limitation will be addressed through the implementation of a comprehensive ADMET screening protocol, enabling a more balanced assessment of the potential drawbacks associated with this mechanism of action while preserving its therapeutic relevance.

For the molecular docking protocols, the protein structures were selected based on several established criteria to ensure the quality and relevance of the docking studies. Primarily, we prioritized structures that are publicly available in the Protein Data Bank webpage with high resolution, as high-resolution structures provide more accurate modeling of binding sites. Additionally, we considered the biological relevance of the structures, selecting those that represent the functional forms of the viral glycoprotein (NiV-G), host receptor EFNB3 and EFNB2, which have been extensively validated in previous research [42,43,44,45]. For protein structures with missing amino acid residues, the models were reconstructed using AlphaFoldV3.0.3 3D software. In parallel, the three-dimensional structures of NiV-G (PDB ID: 2VWD), the attachment glycoprotein in complex with the EFNB3 receptor (PDB ID: 3D12), and the EFNB2 receptor (PDB ID: 2VSK) were retrieved from the Protein Data Bank for subsequent structural and interaction analyses.

### 2.2. Minimum Optimization and Protein Preparation

The chemical structures of the selected flavonoids were first subjected to an initial geometry pre-optimization using Avogadro and Chem3D Professional v17.1 (PerkinElmer) to obtain reasonable starting conformations and reduce unfavorable strain. To further refine the ligand geometries prior to docking, each structure was subsequently optimized with the ORCA quantum chemistry package, ensuring energetically consistent conformers for the subsequent binding analyses [46]. Geometry optimization was carried out using the wB97XD functional together with the 6-311G(d,p) basis set, which provides reliable treatment of dispersion and other non-covalent interactions relevant for protein–ligand recognition. The resulting structures were then exported in .pdb format for downstream docking analyses. For protein preparation, the crystallographic structures were processed by removing solvent molecules and other non-relevant heteroatoms. Hydrogen atoms were then added, and Gasteiger partial charges were assigned before docking in order to maintain a consistent protonation state and charge distribution across all receptor models used in the analysis.

### 2.3. Molecular Docking

Molecular docking was used as the first screening step to identify flavonoids with favorable binding profiles against the selected viral and host targets. After protein structures were prepared and ligand geometries optimized, docking calculations were performed with AutoDockGPU 4.2.6. to evaluate ligand interactions with the ephrin receptors EFNB3 and EFNB2. Docking scores and interaction patterns obtained from these simulations are summarized in Table S1.

Apigenin, used as the reference flavonoid in this study, showed a binding energy of −8.2 kcal/mol with the ephrin receptors, with hydrogen bond interactions involving Pro100 and Asn123 and additional hydrophobic contacts with residues such as Leu101, Phe113, Ile115, and Leu127. The glycosylated derivatives of cynaroside and lonicerin displayed more favorable docking scores, with calculated binding energies of −9.1 and −8.7 kcal/mol, respectively with the EFNB2. Cynaroside formed polar interactions with Lys116, Asn123, and Leu124 together with multiple hydrophobic contacts, whereas lonicerin showed hydrogen bonding with Lys116 and Pro122 and stabilizing nonpolar interactions with Phe113 and Ile115. Similar interaction patterns have been reported in structural and computational analyses of ephrin receptor interfaces and flavonoid–protein complexes [47,48].

Additionally, an analysis of the electronic density within the binding site was performed to improve our understanding of the interactions and possible conformational changes occurring during the binding of flavonoids to the EFNB2 and EFNB3 receptors. This analysis revealed a slight predominance of positive partial charges within the binding cavity. This feature is particularly relevant, as it provides insight into the physicochemical nature of the interactions established between these proteins and the flavonoid ligands. To further complement these findings, the HOMO-LUMO distributions of the flavonoids were examined both before and after binding to the proteins. These results are presented in Figures S1 and S2. The analysis showed a redistribution of the frontier molecular orbitals upon binding, suggesting that the ligands undergo electronic reorganization to better accommodate the topology of the protein binding site. This adaptive behavior was most evident for apigenin, where an increase in HOMO density was observed over the benzene ring when interacting with the EFNB2 receptor. Such redistribution may enhance the ligand’s capacity to participate in stabilizing interactions within the binding pocket.

**Figure 2:**
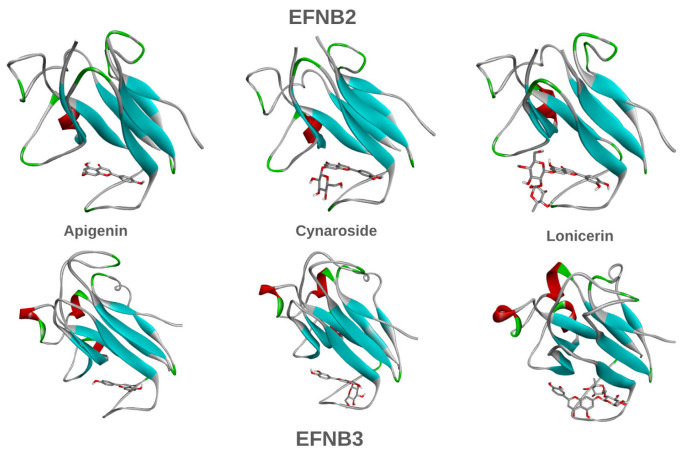
Graphical representation of the complexes formed between the flavonoids and the EFNB2 and EFNB3 receptors.

The differences observed among these flavonoids are consistent with their structural features. Glycosylated compounds provide a broader network of hydrogen bond donors and acceptors, which can favor more extensive interaction patterns within receptor binding regions. In contrast, the aglycone scaffold of apigenin maintains stable but more localized contacts. This behavior has been described previously in comparative docking studies of flavonoids, where sugar substitutions often increase contact density at protein interfaces. In our models, these trends were observed for both EFNB3 and EFNB2, which share a conserved binding surface involved in NiV-G recognition. Across the top-ranked poses, ligand binding at EFNB3 and EFNB2 was dominated by van der Waals contacts, supported by localized hydrogen bonds and dipolar interactions. Residues such as Lys116 appeared repeatedly in the interaction maps, in agreement with structural reports describing this region as part of the ephrin–NiV-G recognition interface [49,50]. A graphical representation of the complexes formed between EFNB2, EFNB3, and the flavonoids is presented in Figure 2.

### 2.4. Pharmacokinetic and Toxicity Properties

The pharmacokinetic, physicochemical, and toxicological profiles of cynaroside, lonicerin and apigenin were assessed using the SwissADME, ProTox v3.0, and pkCSM platforms (https://www.swissadme.ch/, https://tox.charite.de/protox3/, https://biosig.lab.uq.edu.au/pkcsm; accessed in 26 July 2025) [51,52,53]. All relevant molecular descriptors generated by these tools were analyzed, and Table 1 summarizes the key parameters considered most predictive for early-stage drug development. These include physicochemical properties (molecular weight, number of heavy and aromatic atoms, rotatable bonds, hydrogen bond donors and acceptors, lipophilicity [Consensus Log P], and aqueous solubility [Log S, ESOL]), pharmacokinetic attributes (gastrointestinal absorption, blood–brain barrier permeability), drug-likeness criteria (Lipinski’s Rule of Five and any violations), medicinal chemistry indicators (synthetic accessibility), and toxicity endpoints (AMES mutagenicity, oral rat acute toxicity [LD50], chronic toxicity [LOAEL], hepatotoxicity, skin sensitization, and overall toxicity classification) [54,55,56]. All three flavonoids exhibited physicochemical properties consistent with drug-like profiles, with apigenin demonstrating the most favorable overall balance among the evaluated compounds.

Apigenin, a non-glycosylated flavonoid, demonstrated the most favorable predicted oral bioavailability profile among the evaluated compounds. Its relatively low molecular weight (270.24 g/mol), high predicted gastrointestinal absorption, balanced lipophilicity (consensus Log P = 2.11), and full compliance with Lipinski’s rule-of-five criteria support its suitability as an orally active scaffold. All analyzed flavonoids showed moderate predicted aqueous solubility, with Log S values ranging from −3.64 to −3.94.

In silico toxicological assessment, classified cynaroside, lonicerin, and apigenin as non-mutagenic, non-hepatotoxic, and non-sensitizing. Predicted acute oral toxicity values (rat LD_50_) were comparable across the three compounds and fell within ProTox Class 5 (LD_50_: 2000–5000 mg/kg), consistent with low acute toxicity risk. From a medicinal chemistry perspective, apigenin presented the lowest synthetic accessibility score (2.96), indicating lower structural complexity relative to cynaroside (5.17) and lonicerin (6.37), and suggesting more feasible large-scale synthesis. Overall, apigenin combines favorable pharmacokinetic predictions, low-estimated toxicity, and practical synthetic accessibility. Cynaroside and lonicerin, although showing less optimal absorption and higher structural complexity, remain relevant as lead structures due to their interaction patterns with the NiV-G binding interface involving host receptors EFNB2 and EFNB3, supporting their consideration for further optimization and experimental validation.

### 2.5. Molecular Dynamics Simulation

**Figure 3:**
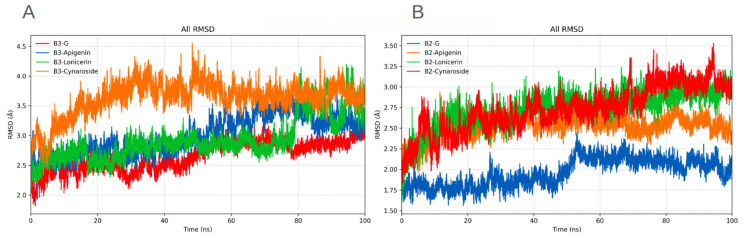
Root mean square deviation (RMSD) profiles of the EFNB3–NiV-G and EFNB2–NiV-G complexes over 100 ns of molecular dynamics simulation. (**A**) Systems involving EFNB3. (**B**) Systems involving EFNB2. The plots depict backbone RMSD values as a function of simulation time for the reference complex and the corresponding flavonoid-bound systems.

Based on their highest docking scores and favorable ADMET profiles, the three top-performing flavonoids were selected for further evaluation of the dynamic stability of their receptor–ligand complexes. Molecular dynamics (MD) simulations were conducted for 100 ns using GROMACS 2024-6 version software [57,58,59], analyzing key structural and energetic parameters, including root mean square deviation (RMSD), root mean square fluctuation (RMSF), radius of gyration (Rg), and binding energy. To better understand how these flavonoids might interfere with the interaction between the viral glycoprotein and its host receptor, two MD simulation setups were employed. The first assessed the stability of each ligand bound to EFNB3 and EFNB2, while the second evaluated the EFNB3–NiV-G and EFNB2-NiV-G complex in the presence of the flavonoids. Upon completion of the simulations, RMSD profiles were analyzed to determine the overall stability of the complexes, as illustrated in Figure 3.

The NiV-G is a type II transmembrane glycoprotein composed of a short N-terminal cytoplasmic tail, a single transmembrane helix, and a C-terminal globular ectodomain responsible for recognition of the EFNB2 and EFNB3 receptors. Binding of NiV-G to these receptors constitutes the initial step of viral entry and triggers conformational activation of the fusion glycoprotein (NiV-F), a critical event required for membrane fusion and subsequent viral entry into host cells.

The root-mean-square deviation (RMSD) is a widely used parameter that measures atomic displacement, particularly within the protein backbone [60]. Sustained low-amplitude RMSD fluctuations throughout the simulation period were interpreted as indicative of preserved structural integrity and stable ligand binding. Such stability supports the reliability of the predicted interactions and provides a robust basis for the rational design of antiviral candidates targeting the early stages of NiV infection [37].

In the molecular dynamics (MD) simulations, the native complexes exhibited trajectories consistent with overall structural stability. The EFNB3–NiV-G complex (Figure 3A, red line) displayed RMSD values ranging from ~2.0 to 3.0 Å over the 100 ns simulation, whereas EFNB2–NiV-G (Figure 3B, red line) showed more restrained deviations (~1.75–2.25 Å), remaining within a narrow fluctuation range throughout the trajectory. These values indicate preservation of the global three-dimensional fold and the absence of large-scale conformational rearrangements. The lower variability observed in EFNB2–NiV-G is biologically consistent with its established role as the primary functional receptor of NiV.

In the presence of flavonoids, modifications in the dynamic profiles were observed relative to the reference complexes. In the EFNB3 system (Figure 3A), the apigenin-bound complex exhibited RMSD values between ~2.75 and 3.5 Å, with a progressive increase after ~40 ns; lonicerin maintained fluctuations around ~2.5–3.0 Å with a transient increase toward the end of the simulation; and cynaroside displayed the largest variations, with peaks reaching ~4.5 Å, suggesting greater conformational perturbation. In EFNB2-based systems (Figure 3B), although RMSD values remained comparatively restrained (~2.2–3.0 Å), deviations from the native system were also evident, indicating ligand-induced modulation of interfacial dynamics.

These variations are particularly relevant because NiV-G acts as a receptor-binding sensor and also serves as a conformational signal transducer to NiV-F. Efficient NiV-F activation requires a stable and geometrically precise interaction between NiV-G and EFNB2/B3. Alterations in the structural dynamics of the interface may affect the temporal persistence, molecular packing, or spatial orientation of critical residues involved in this allosteric communication. In class II viral glycoproteins, even moderate changes in interfacial stability can influence receptor–glycoprotein coupling efficiency and, consequently, the activation of the membrane fusion machinery.

**Figure 4:**
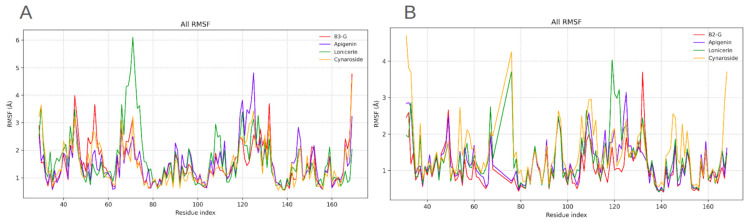
Residue-level flexibility of ephrin receptors in the presence of selected flavonoids. Root mean square fluctuation (RMSF) profiles are shown for (**A**) EFNB3 and (**B**) EFNB2. The plots depict residue-specific backbone mobility across each receptor sequence upon flavonoid binding, highlighting differences in local conformational dynamics between the two receptor systems.

In terms of infectivity, reduced stability or conformational coherence of the NiV-G–receptor complex may result in suboptimal activation of the NiV-F. This could decrease the likelihood of the structural rearrangements required for membrane fusion, ultimately lowering viral entry efficiency. Residue-level flexibility was evaluated through RMSF analysis to determine how flavonoid binding modulates the local conformational dynamics of EFNB3 and EFNB2 (Figure 4).

In the EFNB3-based system (Figure 4A), the native EFNB3–NiV-G complex exhibited moderate backbone fluctuations, with most residues remaining below 2.5 Å and discrete peaks corresponding to solvent-exposed loop regions. However, flavonoid binding altered this profile in a residue-dependent manner. Notably, lonicerin induced a marked increase in flexibility around residues 70–75, whereas apigenin and cynaroside promoted localized increases within the region spanning approximately residues 115–130 and toward the C-terminal segment. This region includes Lys116, a residue structurally implicated in the NiV-G binding interface. Structural studies have demonstrated that Glu533 in NiV-G forms salt bridges with Arg57 and Lys116 in ephrin, and mutations in Glu533 abrogate viral attachment and membrane fusion. These findings highlight the functional importance of this electrostatic interaction network in stabilizing the receptor–glycoprotein complex [61,62,63,64,65].

In this context, increased local mobility around Lys116 could influence the geometry or persistence of these salt-bridge interactions without necessarily disrupting the overall fold of the receptor. Given that salt bridges and hydrophobic core interactions contribute substantially to binding affinity, even moderate dynamic perturbations in this region may affect receptor–glycoprotein coupling efficiency. By contrast, the EFNB2-based system (Figure 4B) displayed a more restrained fluctuation profile. Most residues remained below 2.0–2.5 Å, including the segment corresponding to Lys116, suggesting greater interfacial stability. Although localized mobility increases were observed in certain regions, these were generally less pronounced than in EFNB3. The relative rigidity of EFNB2 is consistent with its established role as the primary entry receptor for NiV, where precise geometric alignment is required to sustain the critical electrostatic interaction with Glu533 of NiV-G and to facilitate subsequent activation of the fusion protein.

**Figure 5:**
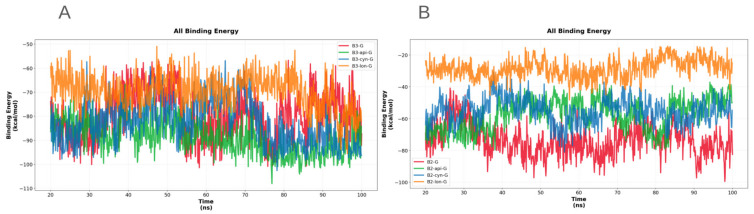
Binding free energy (ΔG) profiles of (**A**) EFNB3 and (**B**) EFNB2 interactions with NiV-G and selected flavonoids over the course of the simulation. ΔG values were calculated throughout the trajectory to evaluate the energetic stability of the complexes and to compare ligand-induced effects on interaction affinity in both receptor systems.

Overall, the RMSF profiles indicate that flavonoid binding may differentially modulate the dynamics of functionally relevant interfacial regions, particularly in EFNB3. While no large-scale destabilization is observed, localized flexibility changes in residues such as Lys116 could subtly influence the fine structural organization of the interaction network that underpins viral attachment and conformational signaling for fusion activation. The Radius of gyration (Rg) was monitored over 100 ns to assess global compactness in both EFNB2–NiV-G and EFNB3–NiV-G complexes, as well as in their corresponding flavonoid-bound systems (Figure S3). Across all trajectories, Rg values fluctuated within relatively narrow ranges, indicating preservation of the overall structural organization of the receptor–glycoprotein assemblies. No evidence of large-scale unfolding or structural collapse was observed in any system. Taken together, the Rg analysis supports that the energetic and interfacial effects described below occur within structurally stable receptor–glycoprotein frameworks. Binding free energies for EFNB3, NiV-G, and the different ligands were estimated using the MM/PBSA approach implemented in GROMACS. In this approach, the binding free energy was estimated as the difference between the total free energy of the protein–ligand complex and the sum of the free energies of the isolated receptor and ligand extracted from the same molecular dynamics trajectory. The calculations were performed using representative snapshots obtained from the production phase of the simulation. Within this framework, differences in calculated binding energy values reflect variations in the strength of intermolecular interactions between the molecular partners. The results of these calculations are presented in Figure 5.

Across all systems analyzed, ΔG values remained negative throughout the evaluated interval of 20 to 100 ns, indicating that complex formation is energetically favorable under all simulated conditions. However, clear receptor- and ligand-dependent differences were observed.

A distinct pattern was observed in the EFNB2 systems presented in Figure 5B. The native EFNB2–NiV-G complex showed strongly negative binding energies, often below −70 kcal/mol, consistent with a robust and stable interaction. In the presence of flavonoids, all EFNB2 systems displayed a systematic shift toward less negative ΔG values compared to the control. This effect was particularly evident in the EFNB2–lonicerin–NiV-G system, which showed the most pronounced reduction in binding energy magnitude among all simulations. Although the interaction remained favorable overall, the energetic change was sustained across the trajectory, suggesting a meaningful attenuation of receptor–glycoprotein stabilization. Apigenin and cynaroside also reduced binding strength relative to the native complex, albeit to a lesser extent. To further validate the robustness of the MM/PBSA results obtained for the flavonoid-EFNB2 systems, replicate molecular dynamics simulations were performed (Figure S4). The calculated binding free energy profiles showed consistent trends across independent trajectories, supporting the reproducibility and reliability of the energetic estimations.

The energetic reductions observed, particularly in the EFNB2 systems in Figure 5B and in the EFNB3–lonicerin-NiV-G system in Figure 5A, may reflect alterations in interfacial complementarity or stability that influence viral attachment efficiency. Although the flavonoids did not completely abolish receptor–glycoprotein association, the attenuation of binding strength, together with the partial disengagement observed for EFNB3 in the presence of lonicerin, indicates that certain compounds can meaningfully interfere with optimal complex stabilization. Considering the central role of EFNB2 as the primary entry receptor for NiV, even moderate reductions in predicted binding affinity may translate into decreased efficiency of viral attachment and subsequent fusion triggering. Collectively, these findings support the notion that flavonoid binding can modulate EFNB–NiV-G interaction energetics in a receptor-dependent manner while preserving overall structural stability.

**Figure 6:**
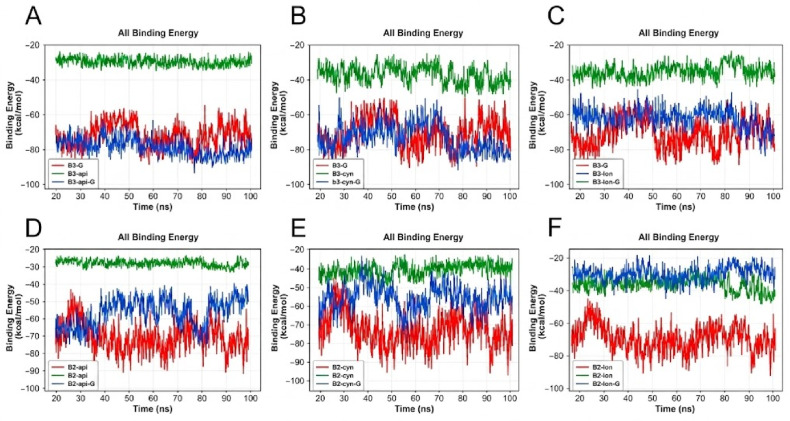
Comparative binding free energy (ΔG) profiles of (**A**–**C**) EFNB3–NiV-G and (**D**–**F**) EFNB2–NiV-G interactions in the presence of selected flavonoids throughout the simulation time. ΔG values were monitored along the trajectory to compare the energetic stability of the complexes and to assess the impact of the compounds on interaction affinity in both receptor systems.

Collectively, the MM/PBSA results suggest that flavonoids may function as energetic modulators of the receptor–glycoprotein interaction in the case of EFNB2 (Figure 6), whereas for EFNB3 their effect appears to be predominantly structural modulation. For EFNB3, the native complex consistently exhibited strongly negative binding energies, typically ranging between approximately −70 and −100 kcal/mol. In the presence of apigenin (Figure 6A), the EFNB3–Apigenin–NiV-G system followed a trajectory closely comparable to the native complex, with only modest deviations and no sustained energetic weakening. Cynaroside (Figure 6B) induced a slightly greater attenuation, particularly during defined temporal windows, where ΔG values shifted toward less negative ranges relative to the control. However, the interaction remained stable overall. In contrast, lonicerin (Figure 6C) produced the most consistent energetic modulation. The EFNB3–Lonicerin–NiV-G complex displayed sustained segments with reduced binding magnitude compared to the native system, aligning with previously noted partial disengagement at the receptor–glycoprotein interface. Although binding remained favorable, the persistent energetic shift suggests a measurable reduction in interfacial stabilization rather than transient fluctuation.

A related yet quantitatively distinct pattern was observed for EFNB2. The native EFNB2–NiV-G complex generally exhibited even more negative binding energies than EFNB3, frequently approaching or surpassing −90 kcal/mol, consistent with its established role as the primary entry receptor for NiV. Upon apigenin binding (Figure 6D), a moderate but stable reduction in ΔG magnitude was observed relative to the native system. Cynaroside (Figure 6E) led to a slightly more pronounced attenuation during specific intervals, though without loss of overall stability. Notably, lonicerin (Figure 6F) again generated the most evident energetic modulation, with sustained reductions in binding strength compared to the EFNB2–NiV-G control. Despite these reductions, ΔG values remained clearly negative, and no trends indicative of global conformational rearrangement were detected.

Taken together, these results indicate that flavonoid binding modulates EFNB–NiV-G interaction energetics in a compound and receptor dependent manner. EFNB2, while energetically more robust in its native interaction, exhibits measurable susceptibility to energetic attenuation, particularly in the presence of lonicerin. However, a pronounced plateau can also be observed, reflecting a sustained cycle of partial interaction and intermittent decoupling between the two proteins. This behavior ultimately converges into a later phase of stabilization, suggesting that the system undergoes a period of dynamic rearrangement before reaching a more energetically consistent interaction state. EFNB3 shows a comparable qualitative trend, with lonicerin again producing the most consistent effect. Importantly, these reductions in predicted binding affinity occur without evidence of large-scale structural destabilization, suggesting that modulation arises from altered interfacial complementarity rather than disruption of receptor integrity. Given the central role of EFNB2 in viral attachment and fusion triggering, sustained moderate reductions in binding energy may have biological relevance by decreasing the efficiency of receptor engagement.

To improve the interpretation of the interactions between the flavonoids and the EFNB2 and EFNB3 receptors, we performed a comparative analysis of the residues involved in these complexes (Figures S5 and S6). Specifically, we examined both the initial interactions identified in the most favorable docking poses and the final interactions observed in the last snapshot of the molecular dynamics (MD) simulations. This comparison provides valuable insight into which contacts persist throughout the simulation time, suggesting the potential relevance of specific residues for maintaining and stabilizing the ligand–receptor complex. In the case of EFNB2, residues Pro100 and Thr99 appear to play a meaningful role in the interaction with the flavonoids, as both residues remain involved in the complexes formed by the two compounds that produced the greatest reduction in interaction energy between EFNB2 and the NiV-G protein. However, after the MD simulations, these hydrogen-bond interactions became less frequent and were largely replaced by van der Waals contacts, indicating a shift in the interaction profile over time.

Regarding EFNB3, the flavonoids showing the most favorable binding energies appear to interact with a slightly different set of residues compared with EFNB2. These differences likely arise from structural and topological variations between the two receptors, which influence cavity shape and residue accessibility. These results are summarized in Table 2 and highlight potential structural determinants that may be useful for guiding the design of compounds with improved receptor selectivity.

Docking studies, combined with ADME–Tox profiling and molecular dynamics simulations, suggest that apigenin and lonicerin are the flavonoids that most consistently modulate the EFNB–NiV-G interface. Rather than functioning as classical steric inhibitors, these compounds appear to exert their effects through interactions with interfacial residues most notably Lys116 accompanied by subtle energetic and conformational changes that may influence the stability of the receptor–glycoprotein complex. In addition, both flavonoids were found to interact with key residues previously implicated in NiV entry, particularly leucine and tryptophan residues located within the G–H loop of EFNB2 and EFNB3. These interactions, illustrated in Figures S5 and S6. The binding profiles reveal a diverse set of interaction types, including hydrogen bonding, van der Waals contacts, and π–π stacking. This variety highlights the capacity of the selected flavonoids to establish multiple stabilizing contacts with critical residues at the interface, reinforcing their potential as modulators of NiV–receptor binding.

Integration of binding free energy (ΔG), radius of gyration (Rg), and root mean square deviation (RMSD) analyses provides a coherent interpretation of the behavior of apigenin, cynaroside, and lonicerin. In the receptor–flavonoid systems, ΔG values indicate thermodynamically stable complexes. Upon formation of the EFNB–NiV-G assembly, however, the presence of flavonoids shifts ΔG toward less negative values relative to native receptor–glycoprotein systems, consistent with reduced interfacial stabilization. This energetic attenuation is most pronounced for lonicerin in both EFNB3 and EFNB2, clearly detectable for apigenin in EFNB2, and moderate but reproducible for cynaroside. Although binding remains thermodynamically favorable, the reduced magnitude of ΔG suggests a measurable weakening of receptor–glycoprotein interaction strength rather than complete disruption.

Receptor-dependent differences further refine this interpretation. The native EFNB2–NiV-G complex exhibits more negative ΔG values than EFNB3, consistent with its established role as a primary entry receptor. Notably, despite its stronger baseline affinity, EFNB2 demonstrates a comparatively greater energetic sensitivity to flavonoid-associated modulation, particularly in the presence of lonicerin. In EFNB3, lonicerin produces a sustained but comparatively less pronounced attenuation, whereas apigenin exerts a milder effect. This pattern suggests that structural conservation of the β-propeller fold and viral binding interface does not translate into identical dynamic or energetic responses. Although EFNB2 and EFNB3 share overlapping viral engagement regions, subtle differences in local residue composition, electrostatic distribution, and intrinsic flexibility may condition how perturbations propagate across the interfacial network. In this context, the greater energetic modulation detected in EFNB2 is interpreted as a subtype-specific dynamic susceptibility rather than structural divergence. Such differential responsiveness may be relevant for future efforts aimed at selectively modulating ephrin receptor engagement.

Rg and RMSD analyses support this interpretation. Across all systems, fluctuations remained within narrow ranges, indicating preservation of overall structural integrity. Minor reductions in Rg and RMSD in flavonoid-bound complexes suggest modest restriction of conformational mobility, without evidence of global destabilization. These observations indicate that the reported energetic effects occur within structurally stable receptor–glycoprotein assembly. Although these observations derive from computational modeling and remain predictive, the consistent pattern of energetic attenuation, coupled with receptor-dependent dynamic responses, supports the hypothesis that selected flavonoids may modulate the EFNB2/B3–NiV-G interface. In view of their recognized polypharmacological behavior and generally moderate binding affinities, these compounds could serve as molecular probes to delineate structural and dynamic determinants of receptor–virus engagement, providing a mechanistic framework that may inform the rational design of more selective and drug-like modulators.

To further strengthen the translational relevance of these findings, it is important to consider experimental strategies that could validate the proposed mechanism and assess both selectivity and safety. As the present study is based on in silico modeling, the hypotheses generated here require confirmation through complementary biochemical and cellular approaches. In this context, future investigations could employ biophysical techniques such as surface plasmon resonance (SPR) or isothermal titration calorimetry (ITC) to quantify flavonoid binding to EFNB2 and EFNB3 and to evaluate potential interference with EFNB–NiV-G complex formation. In parallel, competitive binding or displacement assays would help determine whether these compounds selectively target the virus–receptor interface without broadly disrupting endogenous Eph–ephrin interactions.

At the cellular level, endothelial and neurovascular models represent particularly relevant systems for evaluating potential off-target effects, given the central role of ephrin signaling in vascular and neuronal function. Endothelial barrier integrity assays, including measurements of permeability and junction stability, could be used to assess the impact of flavonoids on vascular homeostasis. Similarly, neuronal or neurovascular cell models may provide insight into potential functional effects in tissues relevant to Nipah virus pathogenesis. Complementary cytotoxicity and selectivity profiling across multiple human cell types would also be essential to determine whether the compounds preferentially modulate virus-related interactions without compromising normal cellular processes. These approaches would help define the therapeutic window and clarify the balance between antiviral potential and physiological safety. The integration of these experimental strategies would provide a robust framework for validating the computational predictions presented in this study, while addressing key questions related to selectivity, mechanism of action, and safety. Such efforts will be essential for advancing flavonoid-based modulation of the EFNB–NiV-G interface from a predictive computational model toward a therapeutically relevant antiviral strategy.

## 3. Materials and Methods

### 3.1. Data Collection

To establish the foundation of our compound-selection workflow, Apigenin was chosen as the initial reference scaffold. This flavonoid served as the baseline scaffold for similarity screening. Using the PubChem structure search tool (https://pubchem.ncbi.nlm.nih.gov), compounds with comparable 2D and 3D chemical features were identified. Subsequently, an extended search was conducted to evaluate additional properties of the retrieved candidates following a Tanimoto similarity analysis. Key selection criteria included evidence of antiviral activity, chemical diversity, and favorable drug-like characteristics. For all selected compounds, SMILES codes and SDF files were obtained for subsequent computational analyses.

Protein structures were selected based on structural quality and biological relevance. Only entries deposited in the Protein Data Bank (https://www.rcsb.org; accessed in 20 June 2025) with resolutions better than 3.0 Å were considered to ensure an accurate definition of binding pockets and interaction interfaces. Experimentally resolved conformations of the NiV-G and its host receptors EFNB2 and EFNB3 were prioritized because of their direct involvement in viral attachment and entry. When relevant regions showed missing residues or unresolved segments, these were reconstructed using AlphaFold-based modeling to obtain complete structures suitable for docking analyses. The three-dimensional coordinates of NiV-G (PDB ID: 2VWD), the NiV-G–EFNB3 complex (PDB ID: 3D12), and the EFNB2 receptor (PDB ID: 2VSK) were retrieved and subsequently prepared for downstream simulations.

### 3.2. Minimum Energy Structures and Protein Preparation

The chemical structures of the selected flavonoids were obtained in digital format and initially pre-optimized using Avogadro 2.0 and Chem3D Professional (version 17.1, PerkinElmer). To ensure accurate ligand conformations for docking studies, further optimization was performed using the ORCA quantum chemistry package. Geometry optimizations employed the WB97XD exchange-correlation functional combined with the 6-311G(d,p) basis set, a method well-suited for capturing non-covalent interactions relevant to molecular docking. Frequency calculations confirmed that all optimized structures corresponded to true energy minima. Hydrogen atoms were added to all ligand structures, and their protonation states were adjusted to neutral conditions at pH 7.4, reflecting physiological environments and facilitating a more accurate simulation of the conditions under which the ligands are expected to function, and Kollman charges were assigned before saving the files in .pdb format for subsequent docking protocols. For receptor preparation, water molecules and any co-crystallized ligands were removed using PyMOL 3.1. Missing hydrogen atoms were added using AutoDock Tools, and the protonation states were assigned to reflect physiological conditions at pH 7.4. Under these conditions, EFNB2 and EFNB3 exhibited net charges of –0.20 and +0.17, respectively. Protonation states were calculated using PDB2PQR (v3.7.1) in conjunction with PROPKA (v3.5.1). Additionally, Gasteiger charges were assigned to ensure an appropriate representation of electrostatic interactions. The prepared receptor structures were then saved in .pdbqt format for molecular docking simulations.

### 3.3. Molecular Docking

To identify and validate potential binding regions for the selected flavonoids on NiV-related targets, a sequential docking workflow was applied. First, putative binding pockets were predicted with the CB-Dock2 server, which combines cavity detection with docking to rank probable ligandable sites based on protein surface topology and pocket geometry. This step was used to define biologically plausible interaction regions for each structure before targeted simulations. Next, blind docking runs were carried out with AutoDock to allow each ligand to sample the full protein surface without spatial restrictions. The convergence between blind docking poses and CB-Dock2 cavity predictions was used to confirm the most consistent binding regions. After this validation step, focused docking calculations were performed with AutoDockGPU to improve conformational sampling and scoring efficiency through GPU acceleration and an ADADELTA-based search scheme. For every ligand–receptor pair, 1000 runs were generated and ranked according to predicted binding energy.

Docking grids were defined individually for each protein using the validated cavity coordinates. For the human cell surface receptor ephrim-B3 (EFNB3; PDB ID: 3D12), the grid center was set at (41, −116, −86) with dimensions of 25 × 25 × 25 Å. For the ephrin-B2 (EFNB2; PDB ID: 2VSK), the docking box was centered at (30, −11, −45) with a size of 24 × 24 × 24 Å. Overall, this stepwise protocol was designed to improve the robustness and reproducibility of the predicted flavonoid–target interactions.

### 3.4. ADME-Tox Prediction

The pharmacokinetic profiles of the selected compounds were assessed using the SwissADME web platform (http://www.swissadme.ch, accesed 20 July 2025). This tool provides a comprehensive evaluation of absorption, distribution, metabolism, and excretion parameters, including gastrointestinal absorption, blood–brain barrier permeability, likelihood of interaction with P-glycoprotein, and potential inhibition of the main cytochrome P450 isoforms (CYP1A2, CYP2C19, CYP2C9, CYP2D6, and CYP3A4). All predictions were carried out using the SMILES representations of the compounds, and the results were systematically recorded for subsequent interpretation. Toxicological properties were predicted using two complementary approaches. pkCSM (http://biosig.unimelb.edu.au/pkcsm/, accesed 26 July 2025) was employed to estimate mutagenicity (AMES test), cardiotoxicity through hERG I and II inhibition, hepatotoxicity, skin sensitization, rat oral LD50, and the maximum tolerated dose in humans. In parallel, ProTox 3.0 (https://tox-new.charite.de/protox_III/, accesed 26 July 2025) was applied to predict acute oral toxicity, assign toxicity classes, identify organ-specific toxicities, and explore relevant toxicological pathways. Data from both platforms were obtained in .csv format and integrated into the analysis to provide a robust overview of the pharmacokinetic and toxicological profiles of the tested compounds.

### 3.5. Molecular Dynamics Simulation

Molecular dynamics (MD) simulations were performed using GROMACS 5.1.3 with the AMBER04 force field [66,67,68,69]. Each protein–ligand complex was placed in a cubic simulation box with a minimum distance of 1.0 nm between the solute and the box edges. The systems were solvated with explicit TIP3P water molecules and electrically neutralized by adding Na^+^ or Cl^−^ counterions as required. No predefined bulk ionic concentration was imposed beyond charge neutralization. Periodic boundary conditions were applied in all three spatial dimensions. Long-range electrostatic interactions were calculated using the Particle Mesh Ewald (PME) method. Covalent bonds involving hydrogen atoms were constrained using the LINCS algorithm, allowing an integration time step of 2 fs.

System preparation was carried out through an automated workflow that generated specific parameter (.mdp) files for each simulation stage. An initial steepest descent configuration was used for ion insertion, followed by energy minimization using the steepest descent algorithm with PME electrostatics. Equilibration was performed in two phases: (i) NVT equilibration for 100 ps (50,000 steps; dt = 0.002 ps) at 300 K using the V-rescale thermostat, and (ii) NPT equilibration for 100 ps at 300 K and 1 bar using the V-rescale thermostat combined with the Parrinello–Rahman barostat under isotropic pressure coupling. Production MD simulations were conducted with a time step of 0.002 ps (2 fs) and a variable number of integration steps defined at runtime. Temperature coupling during production was applied to the entire system. Trajectory, energy, and log files were written every 5000 steps (10 ps). No positional restraints were applied during NVT or NPT equilibration unless specified in the topology. During preprocessing, a tolerance of up to 10 warnings was allowed. Molecular dynamics trajectories were analyzed using descriptive statistical methods over the production phase frames. Time-dependent properties, including root-mean-square deviation (RMSD), root-mean-square fluctuation (RMSF), radius of gyration, solvent-accessible surface area (SASA), temperature, density, volume, and potential, kinetic, and total energies, were extracted using GROMACS. For each parameter, the mean, standard deviation, minimum, and maximum values were calculated and reported. Trajectory stability was assessed based on temporal profiles and fluctuation patterns. Additionally, dynamic cross-correlation matrix (DCCM) analysis was performed to evaluate correlated and anti-correlated motions among residues. When applicable, binding free energies calculated via MM/PBSA were reported as mean ± standard deviation over the sampled frames.

All structural files, docking simulation results, and the scripts used to generate topologies and molecular dynamics trajectories have been provided as Supporting Information. These materials are included to support further analysis, independent verification, and reproducibility of the findings presented in this study. The complete set of raw data has been deposited in a publicly accessible Zenodo repository (https://doi.org/10.5281/zenodo.19101756).

## 4. Conclusions

The present study provides a structured mechanistic framework describing how selected natural flavonoids may modulate the EFNB–NiV-G interaction, a key molecular event governing viral attachment. By integrating DFT, molecular docking, ADME-Tox profiling, molecular dynamics simulations, and MM/PBSA free energy calculations, we characterized the structural and energetic consequences of flavonoid binding within EFNB3 and EFNB2 receptor contexts. The principal advantage of this integrated computational framework lies in its ability to combine complementary layers of molecular evidence to prioritize flavonoid candidates with the potential to interfere with Nipah virus entry. In contrast to approaches that rely solely on docking scores, this workflow incorporates multiple levels of analysis: ligand electronic properties are evaluated through density functional theory (DFT), binding orientation and affinity are assessed via molecular docking, pharmacokinetic and toxicological profiles are examined through ADME-Tox analysis, structural stability is investigated using molecular dynamics simulations, and binding free energies are estimated through MM/PBSA calculations. Consequently, the selected compounds are not only predicted to exhibit affinity for EFNB2/EFNB3 receptors but also to maintain stable interactions under dynamic conditions while displaying favorable drug-like properties.

Across analytical approaches, apigenin and lonicerin exhibited the most consistent modulatory patterns. These compounds engaged interfacial residues, including Lys116 and key leucine and tryptophan residues implicated in NiV entry into host cells and produced measurable attenuation of receptor–glycoprotein binding free energies without inducing global structural destabilization. Reductions in ΔG magnitude, together with modest decreases in RMSD fluctuations and limited shifts in radius of gyration, indicate controlled modulation of interfacial dynamics rather than nonspecific disruption. Importantly, the observed modulation exhibited receptor-dependent characteristics. Although EFNB2 displayed a stronger baseline affinity for NiV-G, it also demonstrated greater energetic sensitivity to the perturbations introduced by the flavonoid ligands. In contrast, EFNB3 showed a more moderate energetic response under similar conditions. This divergence is noteworthy because, despite the two receptors belonging to the same protein family and the compounds sharing a similar initial binding orientation at the start of the MD simulations, their structural behavior differed throughout the trajectory. These findings may serve as an interesting starting point for exploring the structural and physiological distinctions between EFNB2 and EFNB3 and could ultimately prove valuable for improving the selectivity of future ligand designs.

The interaction of flavonoid ligands with ephrin receptors EFNB2 and EFNB3 warrants careful consideration due to the essential physiological roles of these molecules. The localized modulation of the EFNB–NiV-G interface proposed in this study during acute infection may influence the normal function of the receptor, potentially through partial disruption of its native conformation and interference with its physiological interactions with other ligands and protein partners. These effects could contribute to unintended long-term or chronic consequences associated with alterations in ephrin-mediated signaling pathways. Ephrins and their corresponding Eph receptors are key regulators of angiogenesis, cell migration, tissue differentiation, and vascular homeostasis. Consequently, excessive or non-selective pharmacological modulation of these pathways may compromise vascular integrity, potentially impairing neovascularization processes or destabilizing the blood–brain barrier. This concern is particularly relevant given the widespread expression of EFNB2 in endothelial tissues. Moreover, ephrin signaling plays a central role in cell–cell communication and in maintaining tissue organization during both development and adult homeostasis. Disruption of these interactions could adversely affect cellular processes such as proliferation, differentiation, and survival, including those within the immune system. Therefore, despite the favorable pharmacokinetic and safety profiles generally attributed to flavonoids, it is imperative that future experimental and clinical investigations rigorously assess their selectivity and specificity. Special attention should be given to minimizing off-target effects in healthy tissues, ensuring that any inhibitory activity against Nipah virus entry does not come at the expense of essential physiological functions.

While inherently predictive, the convergence of structural stability, dynamic modulation, and free energy attenuation across independent computational approaches strengthens the biological plausibility of these observations. Experimental validation should be carried out using assays that evaluate NiV infection in neuronal cells both in the presence and absence of the selected flavonoids. Furthermore, quantitative binding studies are necessary to assess the stability and affinity of the interactions between flavonoids and ephrin receptors, as well as between ephrin receptors and the NiV-G protein. Such analyses would provide critical insight into how flavonoid binding influences and potentially modulates the virus-receptor interface. Collectively, these findings position apigenin and lonicerin as rational host-targeted scaffolds and provide a structurally grounded basis for advancing flavonoid-based strategies aimed at limiting NiV entry. It is important to highlight that these flavonoids have not previously been proposed as candidates for NiV treatment, positioning this work among the first to explore their potential as scaffolds for modulating this specific viral–host interaction. Beyond identifying candidate molecules, this work contributes a mechanistic perspective that may inform the broader design of interface-modulating antivirals directed at host–virus interaction networks.

## Figures and Tables

**Table 1:** Pharmacokinetic, toxicological, and drug-likeness evaluation of the three selected candidate flavonoids.

Properties		Cynaroside	Lonicerin	Apigenin
**Physico-chemical Properties**	MW (g/mol)	448.38	594.52	270.24
	Heavy atoms (n)	32	42	20
	Arom. Heavy atoms (n)	16	16	16
	Rotatable atoms (n)	4	6	1
	H-bond acceptors (n)	11	15	5
	H-bond donors (n)	7	9	3
**Lipophilicity**	Consensus Log P	0.16	−1.03	2.11
**Water solubility**	Log S (ESOL) (log mol/L)	−3.65	−3.64	−3.94
**Pharmacokinetics**	GI absorption	Low	Low	High
	BBB permeant	No	No	No
**Druglikeness**	Lipinski. Violation (n)	2	3	0
**Medicinal Chemistry**	Synth. Accessibility (score)	5.17	6.37	2.96
**Toxicity**	AMES toxicity	No	No	No
	Oral Rat Acute Toxicity (LD50) (mg/kg)	2.547	2.501	2.45
	Oral Rat Chronic Toxicity (LOAEL) (log mg/day)	4.279	4.626	2.298
	Hepatotoxicity	No	No	No
	Skin Sensitization	No	No	No
	Tox- Level (score)	5	5	5

**Table 2:** Interfacial residues contacts identified by docking and their preservation during molecular dynamics trajectories.

Targets	Ligands	MM/PBSA (kcal/mol)	Hydrogen Bonds/Carbon Hydrogen Bonds	Preserved Interaction
Human cell surface receptor EFNB3 (3D12)	Cynaroside	−43.14	Tyr120, Gln118, Gly168, Glu119	Lys116, Gln118
	Lonicerin	−38.41	Ala97, Thr,114, Asn123, Ser121, Lys115	Pro98, Asn99, Lys116, Asn123, His127
	Apigenin	−28.25	Phe126, Gln118, Ser121	Lys116, Ser121
Human cell surface receptor EFNB2 (2VSK)	Cynaroside	−39.22	Lys116, Gln118, Phe120, Phe129, Leu127, Thr99, Pro100	Lys116, Asn123, Leu124
	Lonicerin	−36.44	Glu119, Pro100, Thr99	Lys116
	Apigenin	−23.94	Phe129, Leu127	Asn123

## Data Availability

The original contributions presented in this study are included in the article/Supplementary Materials. Further inquiries can be directed to the corresponding authors.

## Supplementary Materials

The following supporting information can be downloaded at: https://www.mdpi.com/article/10.3390/ijms27146137/s1.
