# Supplementary material for: Ensemble Docking, MD, and MM/PBSA Identify Flavonoids as Putative Modulators of EFNB2/B3-Nipah Virus G Interaction
Source: Int J Mol Sci. 2026 Jul 9;27(14):6137. doi: 10.3390/ijms27146137 (PMC13410004; doi:10.3390/ijms27146137)
Supplement: Supplementary file 1 [file ijms-27-06137-s001.zip › ijms-4377857-supplementary.pdf]

Ensemble Docking, MD, and MM/PBSA Identify Flavonoids as Putative Modulators of EFNB2/B3-Nipah Virus G Interaction

Carlos Vargas-Echeverría [1,2], Oscar Saurith-Coronell [1,2], Olimpo Sierra-Hernandez [1,2], Juan F. Santos-Rodríguez [2,3], Juan D. Rodríguez-Macías [4,*], José R. Mora [5], José L. Paz [6], Breallan De Jesús Rómero Pájaro [7], German Darío Idarraga Negrete [7], Ricardo Olimpio de Moura [8], Igor José dos Santos Nascimento [8] and Edgar A. Márquez Brazón [2,*]

Supplementary Material

Table S1. Binding affinity values and interaction profiles of selected flavonoids with the host Ephrin receptors EFNB2 and EFNB3.

Targets	Ligands	Binding Energy (kcal/mol)	Hydrogen bonds/ Carbon hydrogen bonds	Other interactions
Human cell surface receptor EFNB3 (3D12)<br>Center (x, y, z): (41, -116, -86)<br>Docking size (x, y, z): (25, 25, 25)	Cynaroside	-9.2	Leu100, Lys116, Gln118	Phe113, Ile115
	Lonicerin	-9.4	Pro98, Asn99, Lys116, Asn123, His127	Leu100, Leu101, Phe113, Pro122
	Fisetin	-8.4	Asn99, Gln118, Ser121, His127	-
	Diosmetin	-8.1	Asn99, Leu101, Gln118, Ser121	Phe113, Ile115
	Luteolin	-8.1	Asn99, Ser121, His127	Phe113, Ile115
	Myricetin	-8.0	Glu119, Tyr120, Ser121, Phe129	Lys116
	Apigenin	-8.5	Lys116, Ser121	Phe113, Ile115
	Quercetin	-8.4	Asn99, Tyr120, Ser121, Asn123	-
	Taxifolin	-8.4	Gln118, Glu199, Asn123, His127	-
	Kaempferol	-8.2	Gln118, Tyr120, Ser121, Asn123	-
Human cell surface receptor EFNB2 (2VSK)<br>Center (x, y, z): (30, -11, -45)	Cynaroside	-9.1	Lys116, Asn123, Leu124	Met83, Leu101, Phe113, Leu127
	Lonicerin	-8.7	Lys116, Pro122	Phe113, Ile115

Docking size (x, y, z): (24, 24, 24)	Fisetin	-8.5	Thr99, Pro100	Met83, Leu101, Phe113, Ile 115, Pro 122, Leu127
	Diosmetin	-8.3	Lys116, Phe120, Asn123	Phe113, Ile115, Pro122, Leu127
	Luteolin	-8.3	Thr114, Lys116, Pro122, Asn123	Met83, Leu101, Phe113, Ile 115, Leu127
	Myricetin	-8.3	Lys116, Asn123	Met83, Leu101, Leu127
	Apigenin	-8.2	Pro100, Asn123	Leu101, Phe113, Ile 115, Leu127
	Quercetin	-8.0	Asn123, Glu128, Phe129	Met83, Leu101, Phe113, Pro 122, Leu127
	Taxifolin	-8.0	Lys116, Asn123, Glu128	Met83, Leu101, Phe113, Pro 122, Leu127
	Kaempferol	-7.9	Phe120, Leu127, Glu128	Met83, Leu101, Phe113, Pro 122

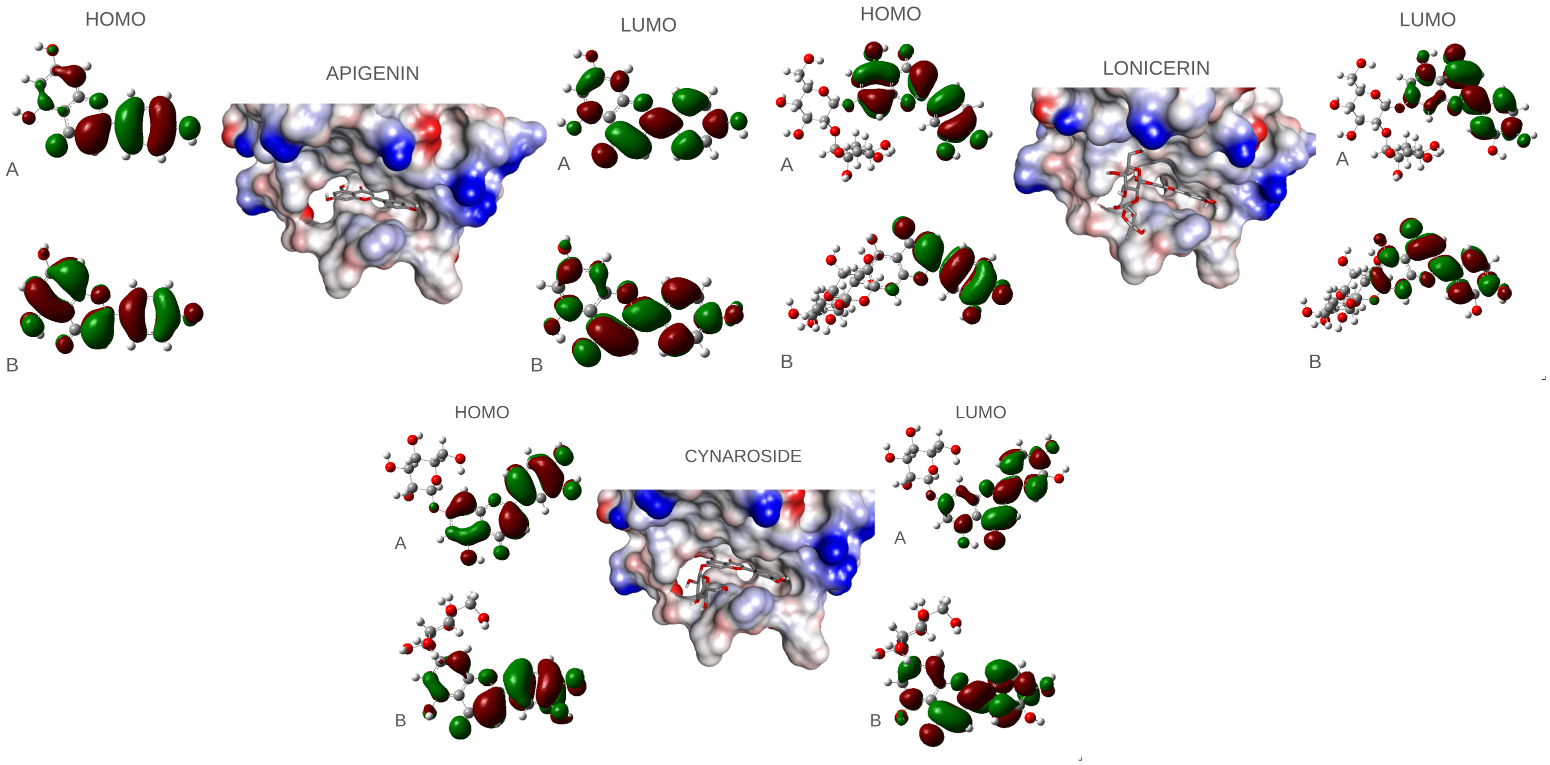

Figure S1. Representation of the electronic density of the binding sitie of the EFNB2 in presence of the flavonoids and HOMO-LUMO representation of the ligands unbound state (A) and bound state (B) the binding with the protein.

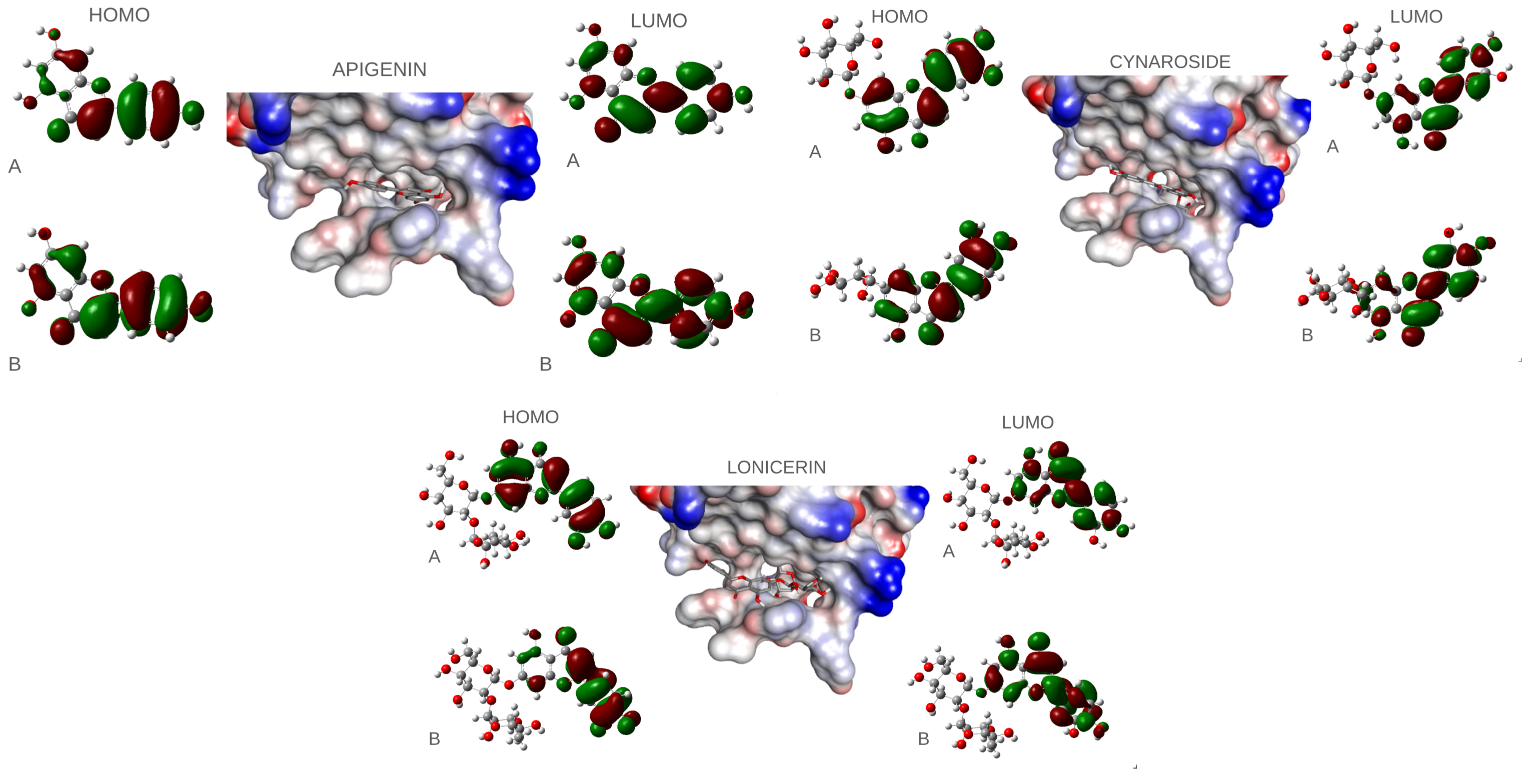

Figure S2. Representation of the electronic density of the binding sitie of the EFNB3 in presence of the flavonoids and HOMO-LUMO representation of the ligands unbound state (A) and bound state (B) the binding with the protein.

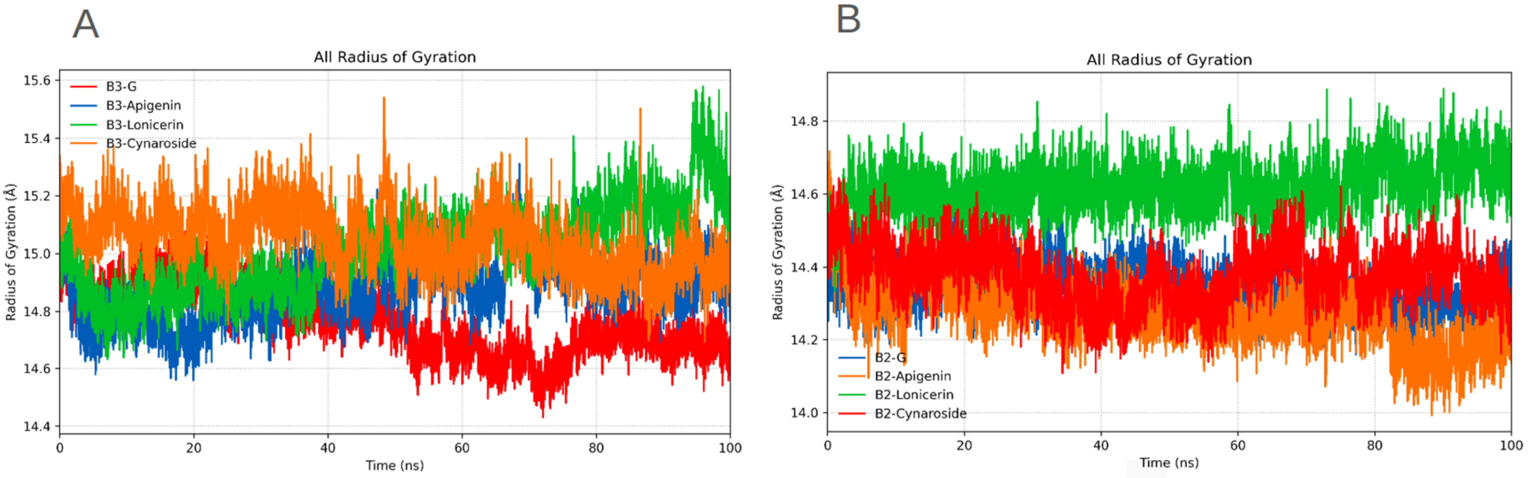

Figure S3. Radius of gyration (Rg) analysis of ephrin receptors in complex with flavonoids and the NiV-G protein. (A) EFNB3-based system. (B) EFNB2-based system. Rg profiles were used to evaluate the overall structural compactness of each complex throughout the simulation, providing insight into potential ligand-induced effects on conformational stability.

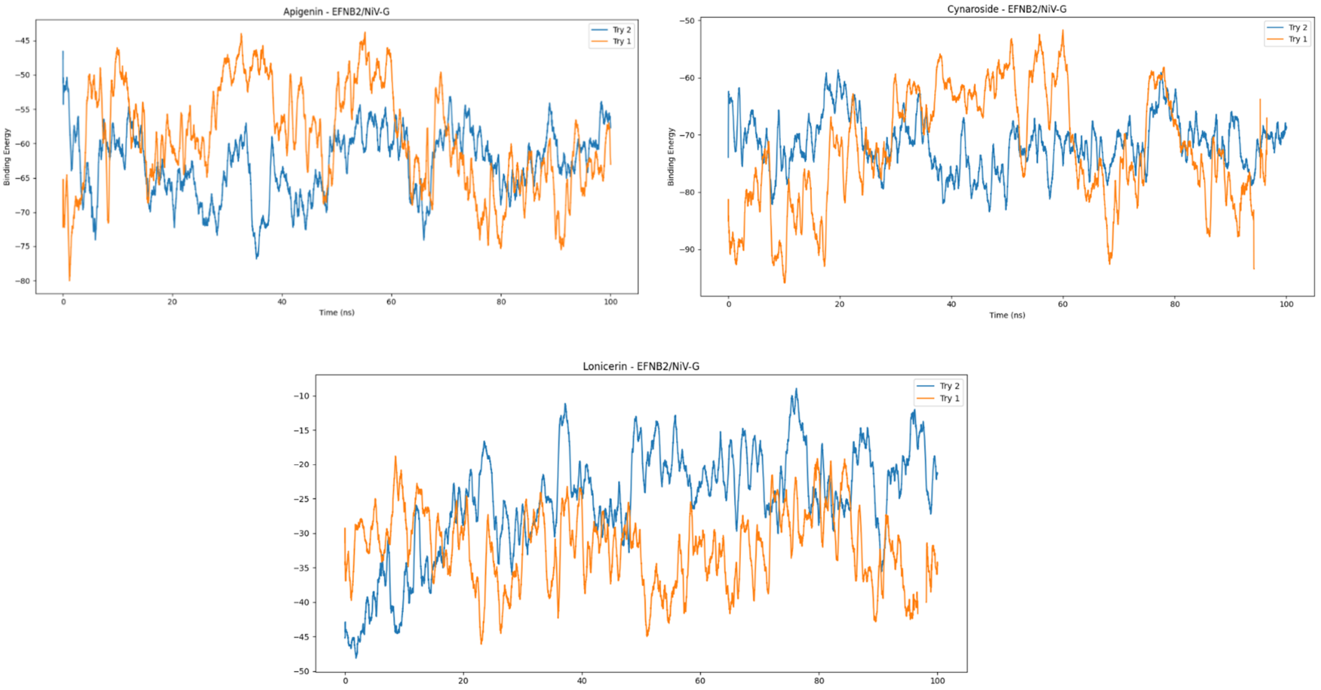

Figure S4. MM/PBSA total binding energy profiles of the EFNB2/NiV-G complexes with apigenin, lonicerin, and cynaroside during 100 ns molecular dynamics simulations. These profiles were used to verify trajectory behavior and the consistency of binding energy trends throughout the simulations.

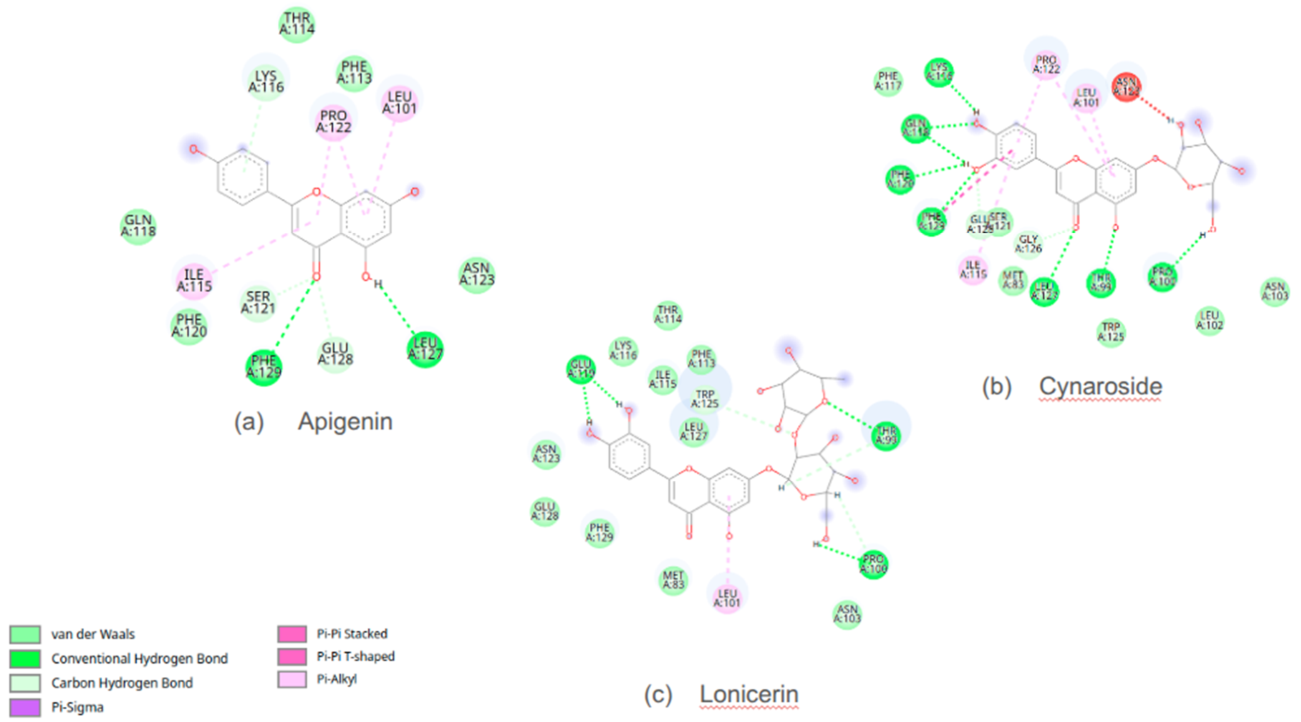

Figure S5. Key interactions of the flavonoids with EFNB2, highlighting the residues that contribute to complex stabilization.

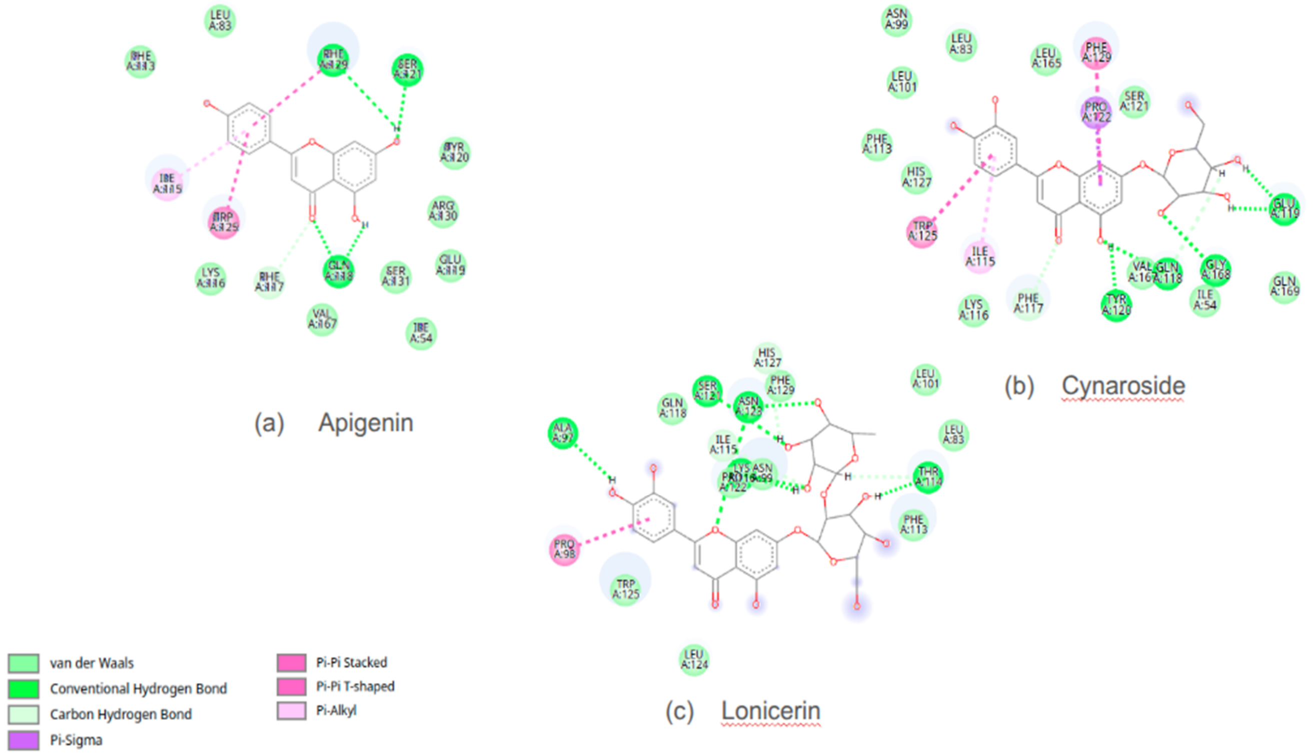

Figure S6. Key interactions of the flavonoids with EFNB3, highlighting the residues that contribute to complex stabilization.
